# Sickle cell disease up‐regulates vasopressin, aquaporin 2, urea transporter A1, Na‐K‐Cl cotransporter 2, and epithelial Na channels in the mouse kidney medulla despite compromising urinary concentration ability

**DOI:** 10.14814/phy2.14066

**Published:** 2019-04-29

**Authors:** Hong Wang, Ryan G. Morris, Mark A. Knepper, Xiaoming Zhou

**Affiliations:** ^1^ Department of Medicine Uniformed Services University of Health Sciences Bethesda Maryland; ^2^ Systems Biology Center NHLBI NIH Bethesda Maryland

**Keywords:** Countercurrent multiplication, NFAT5, SHP‐1, TonEBP, Vasa recta

## Abstract

Sickle cell disease (SCD)‐induced urinary concentration defect has been proposed as caused by impaired ability of the occluded vasa recta due to red blood cell sickling to serve as countercurrent exchangers and renal tubules to absorb water and solutes. However, the exact molecular mechanisms remain largely unknown. The present studies were undertaken to determine the effects of SCD on vasopressin, aquaporin2 (AQP2), urea transporter A1 (UTA1), Na‐K‐Cl co‐transporter 2 (NKCC2), epithelial Na channels (ENaC), aquaporin1 (AQP1), nuclear factor of activated T cells 5 (NFAT5) and Src homology region‐2 domain‐containing phosphatase‐1 (SHP‐1), an important regulator of NFAT5, in the Berkeley SCD mouse kidney medulla. Under water repletion, SCD only induced a minor urinary concentration defect associated with increased urinary vasopressin level alone with the well‐known effects of vasopressin: protein abundance of AQP2, UTA1 and ENaC‐*β* and apical targeting of AQP2 as compared with non‐SCD. SCD did not significantly affect AQP1 protein level. Water restriction had no further significant effect on SCD urinary vasopressin. NFAT5 is also critical to urinary concentration. Instead, water restriction‐activated NFAT5 associated with inhibition of SHP‐1 in the SCD mice. Yet, water restriction only elevated urinary osmolality by 28% in these mice as opposed to 104% in non‐SCD mice despite similar degree increases of protein abundance of AQP2, NKCC2 and AQP2‐S256‐P. Water‐restriction had no significant effect on protein abundance of ENaC or AQP1 in either strain. In conclusion, under water repletion SCD, only induces a minor defect in urinary concentration because of compensation from the up‐regulated vasopressin system. However, under water restriction, SCD mice struggle to concentrate urine despite activating NFAT5. SCD‐induced urinary concentration defect appears to be resulted from the poor blood flow in vasa recta rather than the renal tubules’ ability to absorb water and solutes.

## Introduction

Sickle cell disease (SCD) is an inherited disorder due to a single nucleotide mutation in the *β*‐globin gene at position 6, resulting in a change from the negatively charged glutamic acid to neutral valine in erythrocytes. After erythrocytes release oxygen, the mutated *β*‐globin becomes polymerized, making the red blood cells sickle‐shaped, which are rigid and difficult to pass small vessels. As a result, these cells are occluded and destructed there, leading to ischemic injury of tissues (Becker 2011). Besides *β*‐globin polymerization, SCD also has other hemoglobin abnormalities, for example, *β*‐thalassemia. The CDC has reported that in the United States, SCD affects approximately 90,000–100,000 people, most of whom are African‐Americans (Fujikura 2016). The disease occurs in about 1 in every 500 African‐American births and 1 in every 36,000 (or 1000–1400; the incidence rate is controversial) Hispanic‐American births (Fujikura 2016).

The countercurrent multiplication in the kidney medulla requires synchronizing steady and varied slow blood flow in the vasa recta with varied water and solutes absorption by the renal medullary tubules based on body hydration status. The countercurrent multiplication is essential for urinary concentration, because it establishes hypertonicity and hyperosmolality that drive water absorption in the renal medulla (Burg et al. 2007; Fenton and Knepper 2007; Sands and Layton 2009). However, hypertonicity and hyperosmolality also exacerbate occlusion and destruction of the sickle‐shaped red blood cells in the medulla. It has been proposed that ischemia resulted from red blood cells sickling and congestion in the vasa recta impair both water and solute absorption by the renal medullary tubules and the capacity of the vasa recta to serve as countercurrent exchangers. These impairments lead to decrease of solute accrual in the medullary interstitium, thereby reducing interstitial osmolality (Buckalew and Someren 1974; de Jong and Statius van Eps 1985). Consequently, SCD patients have a defect in urinary concentration, which can appear as early as in infancy (Becker 2011; Nath and Hebbel 2015).

Absorption of water and solutes by the kidney medullary tubules is dependent on vasopressin and numerous water and ion channels and transporters in the region (Fenton and Knepper 2007). Among these channels and transporters, gene knockout studies reveal that water channel aquaporin‐2 (AQP2) and sodium‐dependent potassium and chloride co‐transporter 2 (NKCC2) are among the most critical ones (Fenton and Knepper 2007). AQP2 dictates the apical water permeability of collecting duct principal cells, which is a rate‐limiting step for water absorption (Wilson et al. 2013). NKCC2 plays a major role in active transport of NaCl in the ascending limb of the loop of Henle, leading to rise in the renal medullary interstitial tonicity (Knepper et al. 1999). Besides NKCC2, the epithelial sodium channels (ENaC) in the outer medullary collecting ducts also contribute to absorption of NaCl and urinary concentration (Ecelbarger et al. 2001; Mironova et al. 2015). Further, urea transporters, for example, urea transporter A1 (UTA1), in the inner medulla absorb urea, which contributes to hyperosmolality in the medullary interstitium (Klein et al. 2012). Vasopressin stimulates ENaC activity (Mironova et al. 2015), expression of AQP2, NKCC2 (Ecelbarger et al. 2001; Wilson et al. 2013) and UTA1 (Klein et al. 2012), and phosphorylation and targeting of AQP2 to the apical membrane of the collecting ducts (Inoue et al. 1999). In the human subjects with normal responsiveness of their collecting ducts to vasopressin, urinary excretion of AQP2 correlates with the circulatory levels of vasopressin (Kanno et al. 1995; Elliot et al. 1996). A pathological low blood level of vasopressin results in central diabetes insipidus (Garrahy et al. 2019) with reduced urinary excretion of AQP2 (Saito et al. 1999). Similarly, Brattleboro rats, a model of central diabetes insipidus, have reduced expression of AQP2, UTA1, NKCC2 and ENaC, and urinary concentration defect. Infusion of a vasopressin analog, 1‐deamino‐8D‐arginine‐vasopressin, into the rats increases expression of AQP2, UTA1, NKCC2 and ENaC (Terris et al. 1996; Ecelbarger et al. 2001; Kim et al. 2004; Klein et al. 2012). On the other hand, in various forms of nephrogenic diabetes insipidus, expression of AQP2, UTA1, NKCC2 and ENaC is reduced, but the blood level of vasopressin is usually elevated as a compensatory mechanism (Sands and Bichet 2006; Moeller et al. 2013).

Besides vasopressin, the transcription factor, NFAT5, also known as TonEBP or OREBP, is also critical for urinary concentration, because it controls expression of AQP2 (Lam et al. 2004; Li et al. 2007; Kuper et al. 2012) and UTA1 (Nakayama et al. 2000; Fenton et al. 2004; Lam et al. 2004; Kuper et al. 2012) and osmoprotective genes such as betaine/glycine transporter (BGT1), aldose reductase (AR) and heat shock protein 70 (HSP70) (Burg et al. 2007), which are essential for the renal medullary cells to survive under the hyperosmotic and hypertonic environment (Lopez‐Rodriguez et al. 2004). In the normal rat medulla, NFAT5 is mainly regulated by nucleo‐cytoplasmic distribution in response to different interstitial tonicity or hydration status. A decrease of the renal medullary interstitial tonicity by furosemide reduces NFAT5 nuclear localization and activity (Sheen et al. 2009). On the other hand, water restriction increases NFAT5 nuclear localization as compared with water loading (Cha et al. 2001).

Despite the well‐known fact that SCD patients have urinary concentration defect, the molecular mechanism by which SCD induces the defect is largely unknown. There are a number of SCD mouse models (Nagel 1998). SCD mice we used were generated by knockout of the endogenous globin A and B and knockin of the human transgene containing the mutated globin gene, commonly referred as the Berkeley model (Paszty et al. 1997; Ryan et al. 1997). This model is the best representative of SCD in patients (Paszty et al. 1997). Knockout of globin A alone with knockin of the transgene did not produce SCD, referred as non‐SCD mice, which served as a control. With these mice, we sought to determine whether SCD reduced urinary vasopressin level, protein levels of AQP2, NKCC2, UTA1, and ENaC, and inhibited NFAT5 transcriptional activity, and whether SCD dampened the response of these channels, transporters and transcription factor to water‐restriction.

## Materials and Methods

### Mice

SCD and non‐SCD mice (3‐4 months old, male and female roughly equal) were purchased from The Jackson Laboratory (Stock number: 003342, http://jaxmice.jax.org/strain/003342.html). The mice with global knockout of chloride channels CLC‐K1 were a gift from Dr. Shinichi Uchida (Matsumura et al. 1999). The mice were handled according to the procedures approved by Uniformed Services University and National Heart, Lung and Blood Institute Institutional Animal Care and Use Committee.

### Urinary concentration assay

Mice were placed in metabolic cages (Hatteras Instruments) and acclimated to a water‐replete gelled diet containing 4 g powder food (OpenSource Diet), 0.09 g agar and 5 ml water/20 g body weight/24 h (Mironova et al. 2015) for 48 h. Then, the mice were fed either with the same diet same ration or with a water restricted diet of 4 g powder food, 0.05 g agar and 1 mL water/20 g body weight/24 h for 28 h (Zhou et al. 2014). Urine samples were collected during this period under mineral oil. Urinary osmolarity was measured by freezing point depression with Fiske micro‐osmometer 210.

### Western analysis

The kidney was dissected under a magnifier to isolate outer medulla and inner medulla (including papilla). Tissues were homogenized in 10 mmol/L triethanolamine, pH 7.4 and 250 mmol/L sucrose supplemented with a protease inhibitor tablet (Roche), 2.0 mmol/L NaF and 2.0 mmol/L Na_3_VO_4_. The protein concentrations in homogenates were determined with BCA assay (Pierce). Protein samples (18 *μ*g/lane in most cases) were fractionated in 4–12% Bis‐Tris gel and then transferred to nitrocellulose membranes (Invitrogen) according to the manufacturer's protocol. The membranes were first blocked with Odyssey Blocking Buffer for 60 minutes at room temperature (Li‐Cor) and then incubated with primary antibodies overnight at 4°C. After a brief wash with PBS plus 0.1% Tween‐20, the membranes were incubated with Alexa Fluor‐conjugated secondary antibodies at room temperature for 60 min. Protein expression and phosphorylation were analyzed by an Odyssey Infrared Imager (Li‐Cor). The rabbit antibodies against AQP2, AQP2‐S256‐P, UTA1, AQP1, NKCC2, ENaC‐*α*,* β*, and *γ* were described previously (Fernandez‐Llama et al. 1998; Inoue et al. 1999; Masilamani et al. 2002; Xie et al. 2010). The rabbit anti‐SHP‐1 (SC‐287), NFAT5 (SC‐13035), and actin (SC‐1615) antibodies were purchased from Santa Cruz Biotechnologies. The rabbit anti GAPDH (2118) was bought from Cell Signaling Technologies. The mouse anti SHP‐1 (610125) and rabbit against SHP‐1‐S591‐P (SP‐1531) antibodies were purchased from BD Transduction Laboratories and ECM Biosciences, respectively.

### Urinary vasopressin assay

The urinary vasopressin concentrations were measured with an EIA kit (ADI‐900‐017, Enzo Life Sciences) according to the manufacturer's protocol.

### Immunohistochemistry

The whole kidney was fixed with 10% formalin in neutral buffer (Thermo Scientific) for 72 h, then paraffin‐embedded and sliced at 5 *μ*mol/L each in the university histology laboratory. Immunohistochemistry was performed according to the procedures described in Cell Signaling Technologies website (http://www.cellsignal.com/contents/resources-protocols/immunohistochemistry-protocol-%28paraffin%29/ihc-paraffin). Briefly, tissues were deparaffinized and rehydrated with xylene, ethanol and water sequentially. Antigens were recovered by boiling slides in a microwave in sodium citrate buffer, EDTA buffer, and then Tris‐EDTA buffer. Nonspecific binding was blocked by Odyssey Blocking Buffer at room temperature for one hour. Slides were then incubated with a rabbit anti‐SHP‐1 (SC‐287), NFAT5 (SC‐13035, Santa Cruz Technologies) antibody at a 1:40 dilution or the rabbit anti‐AQP2 antibody described above (1:200 dilution) overnight at 4°C. The primary antibody binding was recognized by an Alexa Fluor 647‐conjugated secondary anti‐rabbit antibody and imaged with a confocal microscope LSM710. AQP2 apical targeting was semi‐quantified by manually counting cells with more apical staining than basolateral staining. NFAT5 nuclear localization was estimated by manually counting cells with increases in nuclear staining. Total 100 cells with AQP2 or NFAT5 positive staining regardless of staining pattern were counted from one randomly selected image per mouse with three mice in total.

### qPCR

The total RNA from the kidney inner medulla was extracted with a RNAzol RT kit (Molecular Research Center) according to the manufacturer's protocol and measured by NanoDrop 8000 (Thermo Scientific). However, cDNAs were synthesized with the High Capacity cDNA Reverse Transcription Kit (Applied Biosystems). mRNAs were quantified with a SYBR Green PCR kit (QuantiFast, Qiagen) in Applied Biosystems 7900HT. The primers for NFAT5, AQP2 and BGT1 were same as before (Moeller et al. 2013). The primers for AR are 5′‐CTATTTCCCACTGGATGCCT‐3′ (Forward) and 5′‐TTTCACCAAACCTTCATCCA‐3′ (Reverse). The amount of 200 ng RNA/reaction was used. 18s rRNA was used to control for the amount of cDNA used in each analysis, but mRNA abundance was not normalized to 18s rRNA. The 18s rRNA primers and probe were purchased from Applied Biosystems. Fold difference in mRNA abundance between conditions (*F*) was calculated, as described previously (de Jong and Statius van Eps 1985).

### Statistical analysis

Results are expressed as mean ± SEM. The reading from the first mouse in the non‐SCD group in Western and qPCR analyses was arbitrated as one and all other results were normalized to this value. Statistical analyses were performed by nonpaired *t* test. The significance of the difference between the effects of water restriction in non‐SCD and in SCD groups was examined by two‐way ANOVA. Multiple comparisons were made with Sidak test. *P* ≤ 0.05 is considered significant.

## Results

### Effect of SCD on urinary concentration

**Figure 1:**
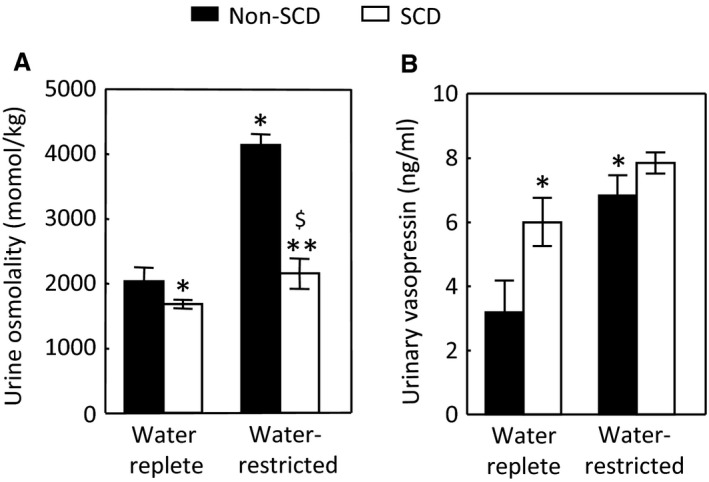
SCD induces urinary concentration defect despite elevation of the urinary vasopressin level. (A) After acclimation to metabilic cages and a water‐replete gel food for 48 h, mice were fed either with the same diet or with a water restricted diet (water reduced by 80%) for 28 h. (B) SCD increases the urinary vasopressin level under water repletion and water restriction only significantly elevates the urinary vasopressin in the non‐SCD group. Urinary vasopressin was measured by an EIA kit. (*P < 0.05 vs. water‐replete non‐SCD, **P < 0.05 vs. water‐replete SCD, ^$^ *P* < 0.05 vs. water‐restricted non‐SCD,* n* = 6 in each group).

With a water‐replete diet, SCD mice were able to maintain urine osmolality that was only 17% below that of non‐SCD mice. However, under the water‐restricted condition, although SCD mice could concentrate urine, the concentration ability was significantly compromised as measured by increase of urinary osmolality (28% vs. 104%, Fig. 1A). We conclude that SCD impairs urinary concentration ability in mice.

### Effect of SCD on the urinary vasopressin level

To determine whether SCD‐induced urinary concentration defect is the central origin, we measured the urinary vasopressin concentrations, indicative of its blood levels. Under water repletion, SCD elevated the mean urinary vasopressin level by 88% as compared with non‐SCD. Water restriction increased the urinary vasopressin level by 114% in the non‐SCD mice but only by 31% in the SCD mice, and the effect in SCD mice was not statistically significant (Fig. 1B). We tried to collect 24 h urine samples under both water replete and restricted conditions, so that we could measure urinary vasopressin excretion in 24 h. But we could only complete the study in two of six SCD mice, because four of these mice played with the food, which blocked the exit of urine into the collection vials, making measurements of 24 h urine output inaccurate. The average of urinary output from these two SCD mice were almost double the mean of urinary output of six non‐SCD mice under water repletion and reduced over 50% following water restriction. Therefore, SCD mice were likely to excrete more urinary vasopressin under water repletion and have less increase under water restriction if the urinary output of vasopressin was expressed in 24 h urine volume. We conclude that SCD induces urinary concentration defect despite increase of urinary excretion of vasopressin.

### Effects of SCD on protein abundance of AQP2, NKCC2 and ENaC‐α, β and γ in the outer medulla and of AQP2, UTA1 and AQP1 in the inner medulla under water‐replete condition

**Figure 2:**
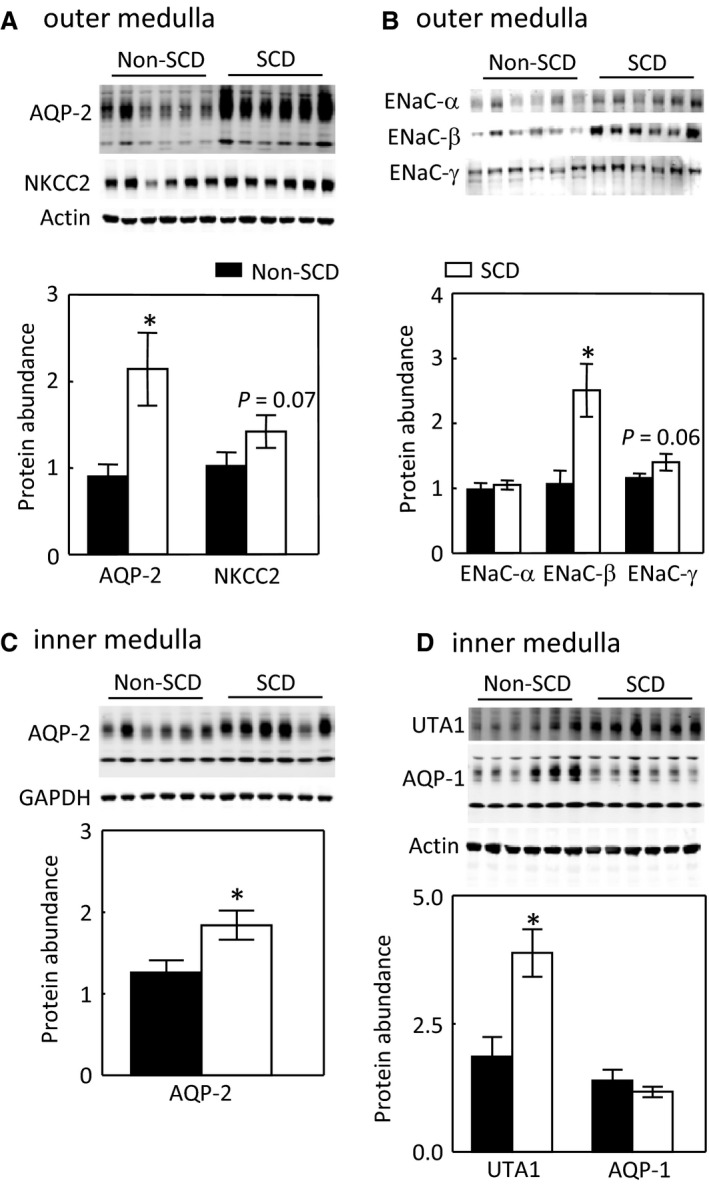
SCD increases protein abundance of AQP2 and ENaC‐*β* in the outer medulla (A–B) and of AQP2 and UTA1 in the inner medulla under water repletion (C–D). Mice were the same ones treated with a water replete diet in Figure 1 (*P < 0.05 vs. non‐SCD group, *n* = 6 in each group).

Having found no evidence suggesting that SCD‐induced urinary concentration defect is due to a decrease of vasopressin in circulation, we turned our attention to the channels and transporters essential to urinary concentration in the kidney medulla to determine whether SCD reduced their expression similarly as observed in the nephrogenic diabetes insipidus. However, to our surprise, in the outer medulla, SCD actually elevated the protein level of AQP2 and had a trajectory to increase protein abundance of NKCC2 (Fig. 2A). ENaC has three isoforms *α*,* β* , and *γ*. SCD significantly increased protein abundance of ENaC‐*β* (Fig. 2B). In the inner medulla, SCD also increased protein abundance of AQP2 as well as UTA1 (Fig. 2C and D). Since AQP1 contributes to urinary concentration (Ma et al. 1998), we also examined the effect of SCD on AQP1 protein abundance in the inner medulla and found that SCD had no significant effect on AQP1 protein level (Figure 2D).

### Effect of SCD on cellular distribution of AQP2 in the inner medulla under water‐replete condition

**Figure 3:**
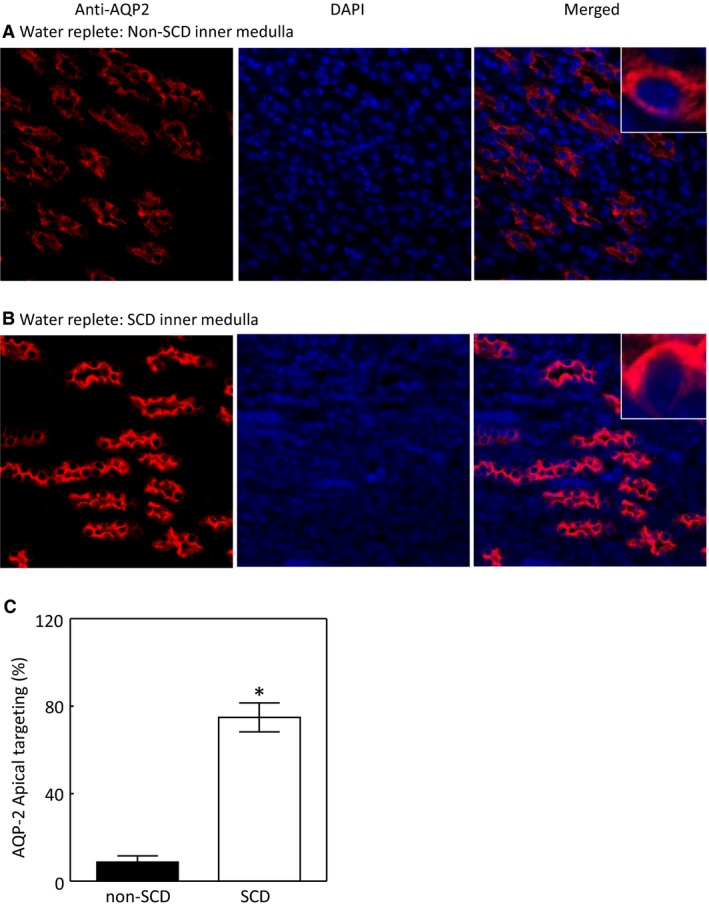
SCD increases the apical targeting of AQP2 in the mouse inner medulla under water repletion. Images were captured under a 40X lens. Representatives of three independent experiments. Apical targeting was defined by increase in apical staining and estimated by manually counting. See Method section for details. (*P < 0.00001 vs. non‐SCD,* n* = 3).

The apical insertion of AQP2 is critical to the apical water permeability of the collecting ducts (Wilson et al. 2013). The un‐expected increase of AQP2 protein abundance prompted us to examine whether SCD trapped AQP2 in the cytoplasm of the inner medullary collecting ducts. A majority of AQP2 was localized in both apical and basolateral regions of the collecting ducts of non‐SCD mice because of the mice were fed with a water replete diet (Figure 3A and C). In contrast, most of AQP2 was in fact concentrated in the apical region of the collecting ducts of SCD mice fed with the same diet (Figure 3B and C). These data indicate that the renal medullary tubules in SCD are responsive to vasopressin stimulation and explain why SCD mice only have slightly defective urinary concentration as long as they are on a water‐rich diet.

### Effects of water restriction on protein levels of AQP2, NKCC2, and ENaC‐α, β, and γ in the outer medulla and protein levels of AQP2, UTA1, and AQP1 and phosphorylation of AQP2 in the inner medulla

**Figure 4:**
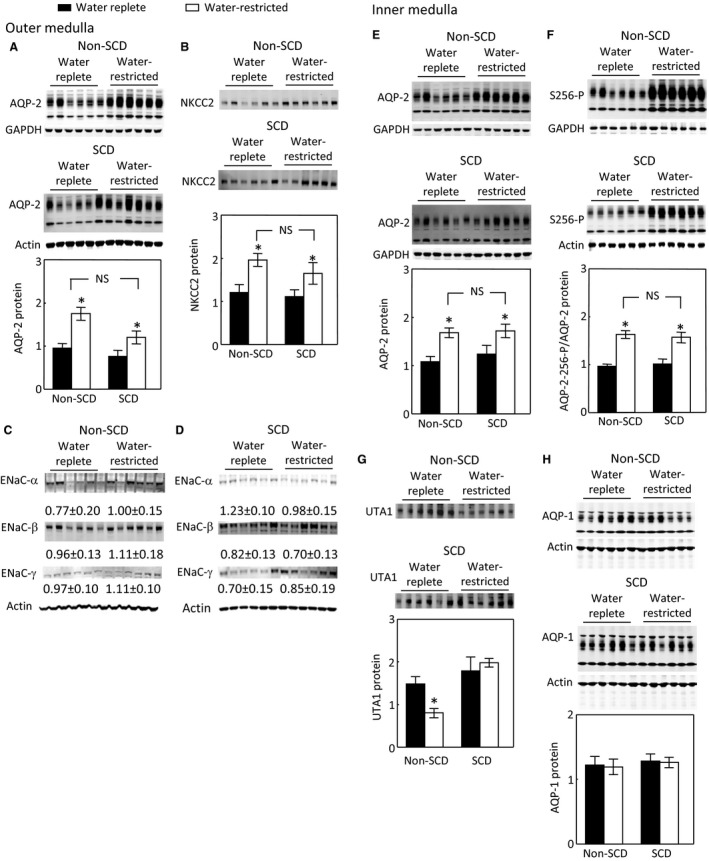
Water restriction significantly increases protein abundance of AQP2 and NKCC2 in the non‐SCD and SCD mouse outer medullas (A and B) and of AQP2 and AQP2‐S256‐P in the non‐SCD and SCD mouse inner medullas (E and F), and these effects are not significantly different between these two strains. Water restriction decreases UTA1 protein abundance in the non‐SCD inner medulla, whereas it has no significant effect in the SCD one (G). Water restriction has no significant effect on protein abundance of ENaC‐*α*,* β* or *γ* in the outer medulla (C and D) or AQP1 protein level (H) in the inner medulla of non‐SCD or SCD group. Mice were treated in the same way as in Figure 1 (*P < 0.05 vs. the corresponding water replete group, *n* = 6 in each group).

Since the SCD mice displayed severe urinary concentration defect (Fig. 1A) and did not significantly elevate the urinary vasopressin level when they were fed with a water‐restricted diet (Fig. 1B), we questioned whether SCD impaired the expression of the channels and transporters in response to the diet. Water restriction for 28 h increased AQP2 protein by 84% and NKCC2 by 62% in the outer medulla of non‐SCD mice (Fig. 4A and B). However, despite lack of a significant effect on the urinary vasopressin level, water restriction still significantly augmented AQP2 protein level by 57% and NKCC2 by 48% in the outer medulla of SCD mice on the top of already increased protein abundance of AQP2 and NKCC2 under water repletion. Moreover, the effects of water restriction on AQP2 and NKCC2 in the SCD mice were not statistically different from those in the non‐SCD mice (Fig. 4A and B). Water restriction had no significant effect on protein abundance of ENaC‐*α*,* β* or *γ* in the outer medulla of either non‐SCD or SCD mice (Fig. 4C–D). Similarly as in the outer medulla, water restriction increased AQP2 protein abundance in a resembling degree in the inner medulla between the non‐SCD and SCD mice (56% vs. 38%, Fig. 4E). Phosphorylation of AQP2‐S256 (AQP2‐S256‐P) is critical for AQP2 function (Wilson et al. 2013). The water‐restricted diet increased AQP‐S256‐P by a similar degree in the non‐SCD and SCD mice (71% vs. 55%, Fig. 4F). However, the effects of water restriction on UTA1 protein abundance were different. Water restriction decreased UTA1 protein abundance in the non‐SCD mice, but had no significant effect in the SCD mice (Fig. 4G). The water‐restricted diet had no significant effect on AQP1 protein abundance in either strain (Fig. 4H). We conclude that SCD increases protein abundance of AQP2 and NKCC2 and phosphorylation of AQP2 in response to water restriction despite lack of significant increase of the urinary vasopressin level, and these effects are not statistically different from the responses from non‐SCD.

### Effect of water restriction on the apical targeting of AQP2 in the inner medulla

**Figure 5:**
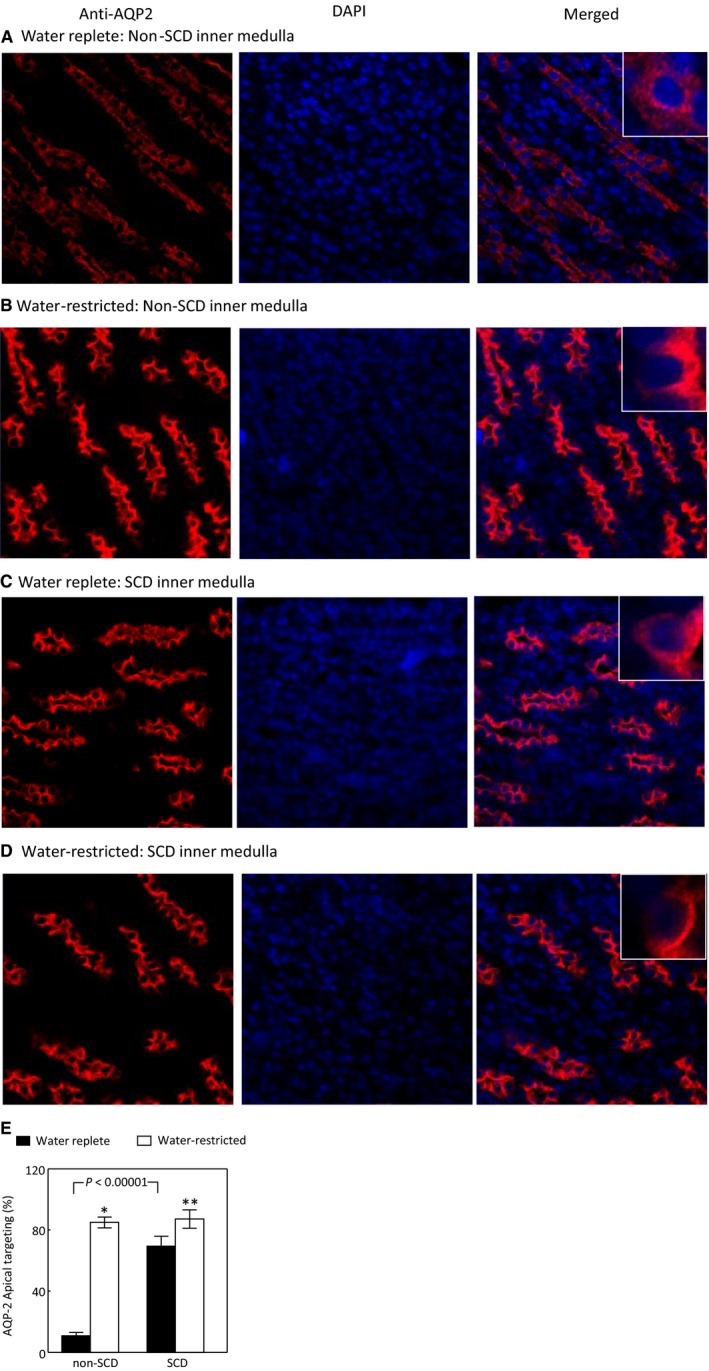
Water restriction increases the AQP2 apical targeting in both non‐SCD and SCD inner medullas. Images were captured under a 40X lens. Representatives of three independent experiments. (*P < 0.00001 vs. non‐SCD under water repletion, **P < 0.05 vs. SCD under water repletion, *n* = 3).

Despite impressive increases in NKCC2 and AQP2 protein levels in the SCD mice following water restriction, the urinary osmolality only rose modestly (Fig. 1A). Thus, we examined whether SCD altered AQP2 apical localization following exposure to the water‐restricted diet. Water restriction significantly increased AQP2 apical targeting in the inner medullary collecting ducts of the non‐SCD mice as expected (Fig. 5A, B and E), but also increased AQP2 apical targeting in the same region of the SCD mice (Fig. 3C–E).

### Effects of SCD on NFAT5 and expression of NFAT5‐targeted genes in the inner medulla under water‐replete condition

**Figure 6:**
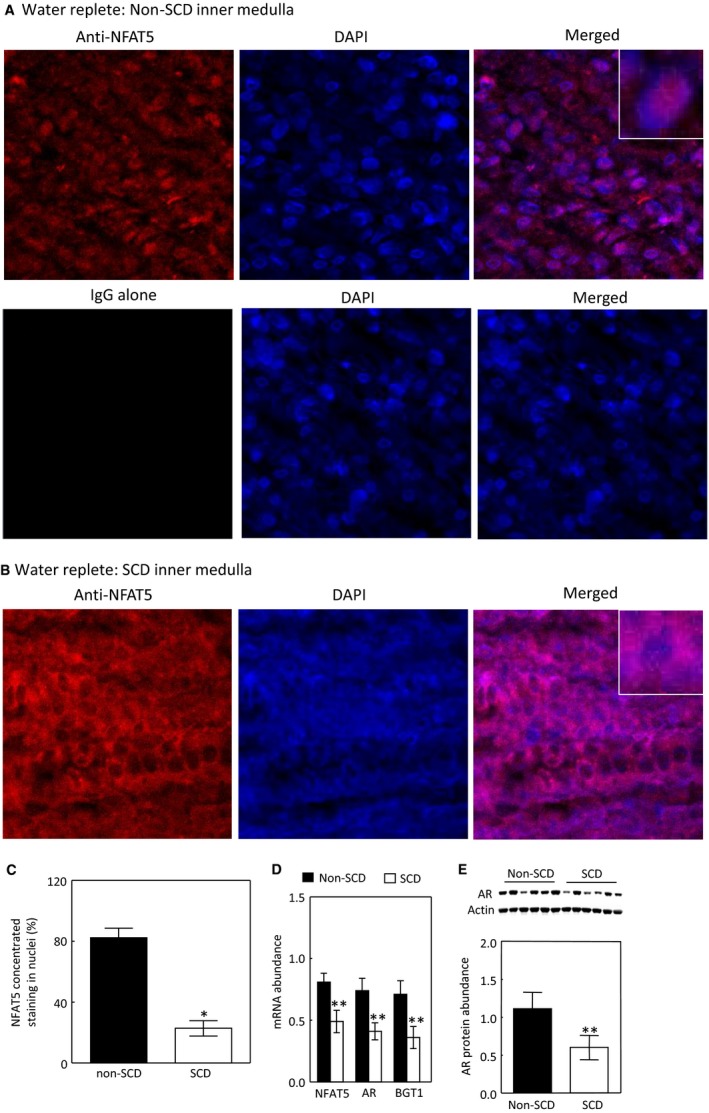
SCD reduces nuclear localization of NFAT5 under water repletion (A–C). Representatives of three independent experiments. NFAT5 nuclear localization was defined by increase in concentrated nuclear staining and estimated by manually counting. See Method section for details. (C, *P < 0.00001 vs. non‐SCD,* n* = 3). SCD reduces mRNA abundance of NFAT5 and its targeted genes: AR and BGT1 (D, **P < 0.05 vs. non‐SCD,* n* = 4 for non‐SCD and *n* = 6 for SCD) and the protein abundance of AR (E, **P < 0.05 vs. non‐SCD,* n* = 6 in each group).

The robust elevation of NKCC2 and AQP2 protein levels upon exposure to water restriction in the absence of significant increase of urinary vasopressin in SCD mice led us to examine the NFAT5 pathway, because NFAT5 has been shown to control AQP2 and UTA1 expression independent of vasopressin (Nakayama et al. 2000; Kasono et al. 2005; Hasler et al. 2006; Gajghate et al. 2009). We first examined the NFAT5 pathway under water repletion. Under this condition, a majority of NFAT5 was localized in the nuclei in the inner medulla of non‐SCD mice as defined by concentrated nuclear staining of the NFAT5 antibody (Fig. 6A and C). SCD reversed this pattern (Fig. 6B and C). SCD also significantly decreased mRNA abundance of NFAT5 and its targeted genes AR and BGT1 (Fig. 6D), and protein abundance of AR (Fig. 6E). The protein abundance of BGT1 was not measured due to lack of a good antibody against it. We were not able to measure NFAT5 protein abundance from the SCD mice, because it was degraded in storage at ‐80^0^ C for some reason, whereas NFAT5 protein from the non‐SCD mice were stable under the same condition. We conclude that under water repletion, SCD decreases NFAT5 nuclear localization as compared with non‐SCD, which leads to the reduced NFAT5 transcriptional activity.

### Effects of water restriction on NFAT5 nuclear localization and expression of NFAT5‐targeted genes in the inner medulla

**Figure 7:**
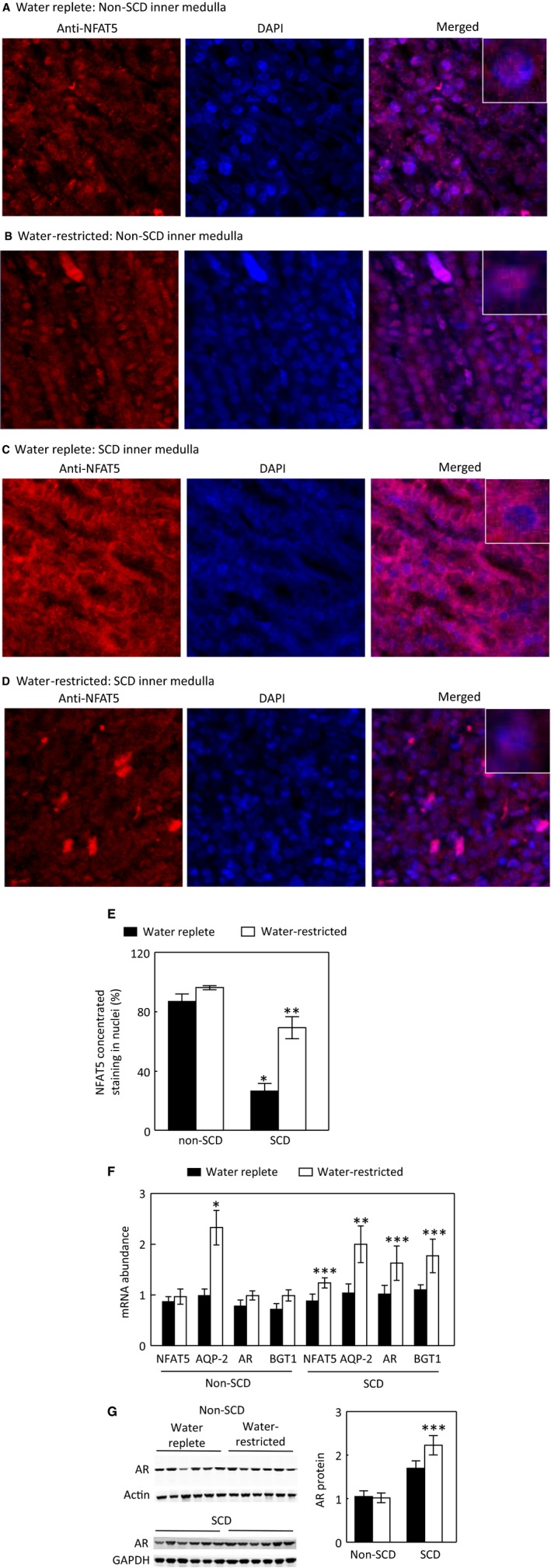
Water restriction for 28 h increases NFAT5 nuclear localization in the inner medulla of SCD mice, whereas its effect in the non‐SCD mice is not significant (A–E). Representatives of three independent experiments. (E, *P < 0.00001 vs. non‐SCD under water repletion, **P < 0.001 vs. SCD under water repletion, *n* = 3). Water restriction for 28 h only significantly elevates AQP2 mRNA level in the non‐SCD group, but significantly increases mRNA abundance of NFAT5, AQP2, AR and BGT1 in the SCD group (F, *P < 0.00001, **P < 0.001, ***P < 0.05 vs. the water replete group, *n* = 6 for the water replete group and *n* = 4 for the water‐restricted group). Water restriction increases the protein abundance of AR in the SCD inner medulla (G, ***P < 0.05 vs. the SCD water replete group, *n* = 6 in each group).

We then examined the effect of water restriction on the NFAT5 pathway. The effect of water restriction on NFAT5 nuclear localization in the non‐SCD mice was not significant by immunohistochemistry staining, since a majority of NFAT5 was already present in nuclei under water‐replete condition (Fig. 7A, B and E). Water restriction had no significant effect on mRNA abundance of NFAT5, AR or BGT1 (Fig. 7F), or AR protein abundance either (Fig. 7G), but increased AQP2 mRNA abundance in these mice (Fig. 7G). In contrast, water restriction increased NFAT5 nuclear accumulation (Fig. 7C–E) and mRNA abundance (Fig. 7F) in the SCD mice. These effects were accompanied with increases of mRNA abundance of AQP2, AR and BGT1 (Fig. 7F) and AQP2 and AR protein abundance (Figs. 4E and 7G). We conclude that water restriction has no significant effect on activation of NFAT5 in the non‐SCD mouse inner medulla, because NFAT5 is already activated in the region, but significantly activates NFAT5 in the inner medulla of SCD mice.

### Effects of SCD and water restriction on SHP‐1

**Figure 8:**
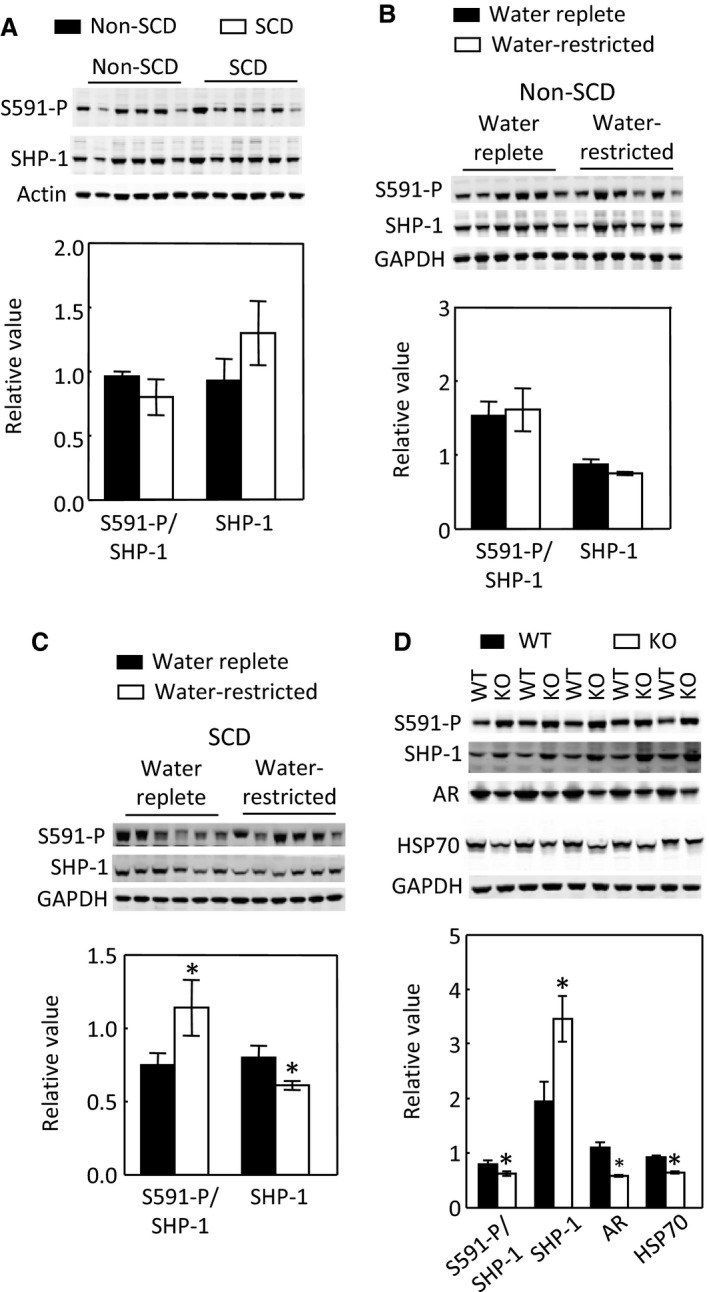
SCD has no significant effect on SHP‐1‐S591‐P or SHP‐1 protein abundance in the inner medulla (A, *n* = 6 in each group). Water restriction has no significant effect on SHP‐1‐S591‐P or SHP‐1 protein abundance in the non‐SCD inner medulla (B, *n* = 6 in each group), but significantly increases SHP‐1‐S591‐P and decreases protein level of SHP‐1 in the SCD inner medulla (C, *n* = 6 in each group). Knockout of ClC‐K1 channels reduces SHP‐1‐S591‐P and increases protein abundance of SHP‐1 associated with reduced protein levels of AR and HSP‐70 in the inner medulla (D, *n* = 5 in each group).

SHP‐1 inhibits NFAT5 by trapping it in the cytosol in HEK293 cells (Zhou et al. 2010) and potentially in the kidney inner medulla (Zhou et al. 2014). To find a clue to how SCD inhibited and water restriction activated NFAT5 in the SCD mouse inner medulla, we measured the effects of SCD and water restriction on the SHP‐1 protein level and inhibitory phosphorylation of serine 591 of SHP‐1 (SHP‐1‐S591‐P). Under water repletion, SCD had no significant effect on SHP‐1‐S591‐P or SHP‐1 protein abundance (Fig. 8A). In parallel to lack of effect of water restriction on NFAT5 in the non‐SCD mice, but activating NFAT5 in the SCD mice, water restriction had no significant effect on SHP‐1‐S591‐P or SHP‐1 protein abundance in the non‐SCD mice (Fig. 8B), but significantly increased SHP‐1‐S591‐P and reduced protein abundance of SHP‐1 in the SCD mice (Fig. 8C). The ClC‐K1 chloride channels are essential for the kidney to effectively concentrate urine. Knockout of ClC‐K1 channels reduces the renal inner medullary interstitial tonicity, resulting in sever urinary concentration defect (Matsumura et al. 1999). As an additional test, we found that knockout of ClC‐K1 channels decreased SHP‐1‐S591‐P and increased SHP‐1 protein abundance, associated with reduced protein abundance of AR and HSP‐70 (Fig. 8D). We conclude that water restriction‐induced activation of NFAT5 is associated with inhibition of SHP‐1.

### Effects of SCD and water restriction on SHP‐1 cellular distribution in the inner medulla

**Figure 9:**
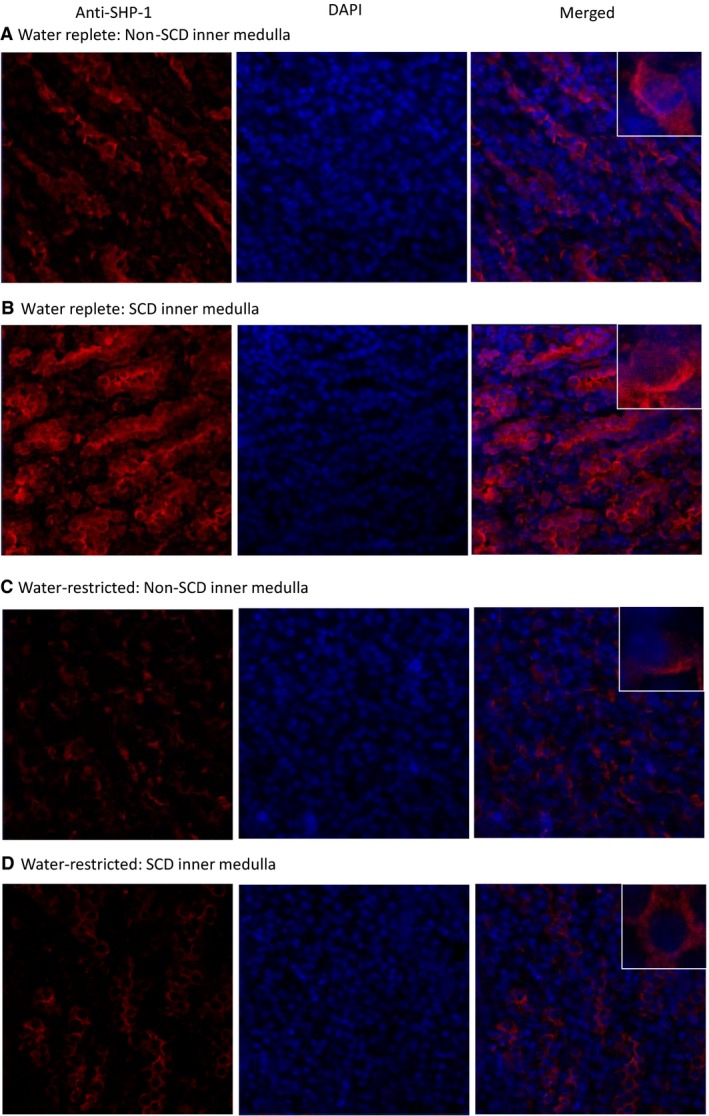
Water restriction for 28 h reduces nuclear localization of SHP‐1 in both non‐SCD and SCD inner medullas, but with different patterns. A reduction in water supply localizes more SHP‐1 to a concentrated area in the cytoplasm in the non‐SCD mice (A and C), whereas it localizes SHP‐1 from nuclei to the cytoplasm without a particularly concentrated area in the SCD mice (B and D). Representatives of three independent experiments.

When the mice were fed with the water‐replete diet, SHP‐1 was distributed in both cytosolic and nuclear compartments with more SHP‐1 localized to the apical region in both non‐SCD and SCD mice (Fig. 9A and B). A reduction in water consumption decreased nuclear presence of SHP‐1 in both types of mice, but with different patterns. Water restriction dislocated more SHP‐1 to the apical region in the non‐SCD mice (Fig. 9C), whereas it expelled SHP‐1 from nuclei to the peri‐cytoplasmic region without a particularly concentrated area in the SCD mice (Fig. 9D). Whether these two patterns have a role in regulation of NFAT5 activity is unknown.

### Effect of SCD on the size of papilla

**Figure 10:**
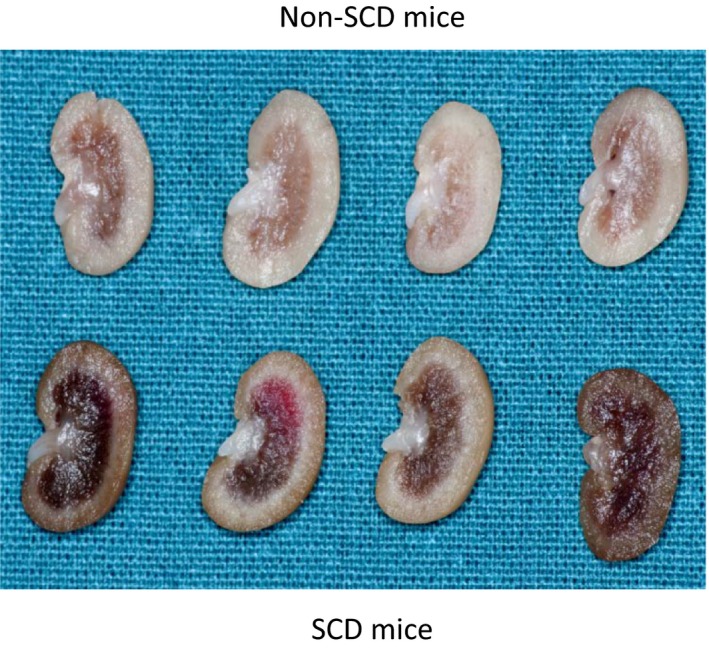
SCD has no obvious effect on the size of papilla except in one mouse, but increases congestion of red blood cells in the outer medulla. Mice were allowed free access to food and water. The kidney was fixed in 10% formalin overnight, then sectioned and photographed.

Although water restriction activated NFAT5 and increased AQP2 in the inner medulla of SCD mice, it did not elevate urinary osmolality to the same degree as it did in non‐SCD mice. One possibility is that the SCD mice may have small papilla, which reduce power of urine concentration, since SCD was shown to induce renal papillary necrosis (Henderickx et al. 2017). Thus, we examined the effect of SCD on the size of the papilla. SCD increased occlusion of red blood cells in the outer medulla as expected, but did not cause obvious reduction in the papilla size except for in one mouse (Fig. 10).

## Discussion

### Uniqueness of SCD‐induced urinary concentration defect

Unlike central or nephrogenic diabetes insipidus, SCD‐induced urinary concentration defect is unique, because it is accompanied with a high level of urinary vasopressin, indicative of a high level of circulatory vasopressin (Fig. 1B), and increased protein levels of AQP2, ENaC‐*β* and possible NKCC2 in the outer medulla, and protein abundance and apical targeting of AQP2 and UTA1 protein abundance in the inner medulla (Figs. 2 and 3). Then, why do SCD patients and mice have urinary concentration defect? The current theory is that the defective urine concentration in SCD is caused by ischemia insult due to congestion of red blood cells in the vasa recta, resulting in impairing both water and solute absorption by the renal medullary tubules and the capacity of vasa recta to serve as countercurrent exchangers (Buckalew and Someren 1974; de Jong and Statius van Eps 1985; Nath and Hebbel 2015). Although direct functional evidence is lacking, the increased protein abundance of AQP2, UTA1, ENaC‐*β* and possible NKCC2, and apical targeting of AQP2 under the water replete condition (Figs. 2 and 3), and no statistical difference in expression of AQP2 and NKCC2 protein levels and in AQP2‐S256‐P and AQP2 apical targeting in response to water restriction from the non‐SCD mice (Figs. 3and 4) indicate that the kidney medullary tubules of SCD mice preserve the ability responding to vasopressin and water restriction. Therefore, the defective urinary concentration in SCD is not resulted from the lack of ability of the medullary tubules to absorb solutes and water. Rather, our findings point to the poor blood flow in vasa recta from red blood cell sickling (Fig. 10) that seems the critical factor, because it impairs countercurrent multiplication, leading to inadequate hypertonicity and hyperosmolality in the renal medullary interstitium, thus diminishing the power that drives urinary concentration. Consistent with this argument is a clinical study showing that the anti red blood cell sickling medication hydroxyurea improves urinary concentration in infants (Alvarez et al. 2012). However, the information concerning how SCD‐induced vascular changes affect urinary concentration is scarce. It is noteworthy that the non‐SCD mice have similar urinary concentration ability as C57BL/6 mice based on our previous study (Zhou et al. 2012). Urinary osmolality rises most within the first 24 h water restriction. Water restriction for 72 h increases the urinary osmolality from 2201 mosmol/kgH_2_O to 4780 mosmol/kgH_2_O in the C57BL/6 mice (Zhou et al. 2012). With the same diets, water restriction for 28 hours increased the urinary osmolality from 2041 mosmol/kgH_2_O to 4143 mosmol/kgH_2_O in the non‐SCD mice (Fig. 1A).

### Under water repletion SCD mice attempt to maintain urinary concentration ability by up‐regulation of vasopressin, whereas under water restriction SCD mice struggle to concentrate urine despite activating NFAT5

Under water repletion, the SCD mice excreted at least 88% more vasopressin from urine than the non‐SCD mice (Fig. 1B). This came along with the well‐known effects of the hormone: increases of expression and apical targeting of AQP2 and of UTA1 protein level. These results explain why SCD mice only had a minor defect in urinary concentration when a water‐rich diet was provided (Fig. 1A). Water restriction had no additionally significant effect on urinary excretion of vasopressin from the SCD mice (Fig. 1B), because SCD already maximized secretion of vasopressin and maxed out the response of urinary concentration‐related transporters and channels in the renal tubules to vasopressin. In support of these arguments are the observation showing that administration of vasopressin fails to correct urinary concentration defect in pediatric SCD patients (Mc et al. 1953). Despite lack of significant effect on the urinary vasopressin, water restriction still significantly increased AQP2‐S256‐P (Fig. 4F) associated with increase of AQP2 apical targeting (Fig. 5). Protein kinase A (PKA) and serum glucocorticoid‐dependent kinase (SGK) involve phosphorylation of S256 (Bradford et al. 2014). PKA and SGK are up‐regulated not only by vasopressin (Bradford et al. 2014), but also by hypertonicity (Waldegger et al. 1998; Ferraris et al. 2002). It is possible that water restriction‐induced hypertonicity activates PKA, SGK and even some other kinases in the medulla of the SCD mice, leading to increases of AQP2‐S256‐P and AQP2 apical targeting.

When the mice were treated with the water replete diet, a majority of NFAT5 was localized in the cytosol in the inner medulla of SCD mice as estimated by only 26.5% of cells having concentrated NFAT5 nuclear staining as opposed 87% of cells having similar staining in the non‐SCD inner medulla (Fig. 6). This distribution pattern was associated with reduced mRNA abundance of NFAT5, AR and BGT1 (Fig. 6D) and protein abundance of AR (Fig. 6E). Since tonicity is a primary regulator of NFAT5 in the kidney medulla (Sheen et al. 2009), the reduced NFAT5 activity reflects the predicted relatively low hypertonicity in the region probably caused by impaired countercurrent multiplication due to congested vasa recta. On the other hand, this low interstitial tonicity enabled water restriction to activate NFAT5, leading to increasing nuclear accumulation of NFAT5 (Fig. 7C–E), and abundance of NFAT5 mRNA (Fig. 7F) with resultant increases of expression of AQP2 in addition to increases of the well‐known osmoprotective genes BGT1 and AR (Fig. 7F and G). Thus, SCD mice tried to concentrate urine by an NFAT5‐dependent mechanism in response to water restriction. Precedent examples of vasopressin‐independent urinary concentration have been repeatedly demonstrated in Brattleboro rats. Water restriction increases urinary osmolality associated with increase of NKCC2 expression and leaves AQP2 protein level unchanged in the rat medulla (Michimata et al. 2003). Feeding the rats with 0.5% NaCl water elevates protein abundance of AQP2 and urinary osmolality (Li et al. 2006). However, despite activation of NFAT5, the SCD mice only managed to concentrate urine by 28% as opposed by 104% by the non‐SCD mice (Fig. 1A). This defect is probably because the congested vasa recta prevent water restriction from raising the tonicity in the medullary interstitial fluid of SCD mice as high as that of non‐SCD mice, thus the driving force for concentrating urine is compromised.

In order to observe the increase of UTA1 protein level by vasopressin, the Brattleboro rats need to be infused with the hormone for 12 days, whereas infusion for 5 days actually decreases UTA1 protein level (Klein et al. 2012). This long‐term effect of vasopressin on UTA1 protein abundance may explain how SCD elevated UTA1 protein level, because SCD, a chronic disease, increased the urinary vasopressin level. Dehydration of C57BL/6 mice for 24 hours reduces UTB protein level (Wang et al. 2016). Water restriction of the non‐SCD mice for 28 hours also had a similar effect on UTA1 protein abundance (Fig. 4G). However, water restriction of the SCD mice for 28 hours did not decrease UTA1 protein abundance (Fig. 4G). A likely explanation is that water restriction also activated NFAT5, which increased expression of UTA1, offsetting the effect of water restriction.

SCD increases glomerular filtration rate (GFR) in children (de Paula et al. 2013) and a mouse model (Bank et al. 1996) and renal blood flow in patients (Etteldorf et al. 1955; Hatch et al. 1970), whereas it actually decreases the mean renal blood velocity in a mouse model (Bonnin et al. 2008). It has been shown that a reduction in GFR is accompanied with a rise in urinary osmolality following water restriction in Brattleboro rats (Valtin and Edwards 1987) and nephrogenic diabetes insipidus patients (McConnell et al. 1977). Whether a perceived decrease in GFR under water restriction in SCD mice contributed to the increase of urinary osmolality cannot be ruled out.

Under the water‐replete condition, a majority of NFAT5 was localized in nuclei in the inner medulla of non‐SCD mice (Fig. 7A and E), thanks to high renal inner medullary tonicity. We found no evidence supporting that water restriction increased NFAT5 activity, because water restriction did not significantly augment NFAT5 nuclear localization (Fig. 7A, B and E), mRNA abundance of AR and BGT1 or AR protein abundance in these mice (Fig. 7F). The increased AQP2 mRNA and protein levels following water restriction is resulted from increased vasopressin effect as repeatedly demonstrated in various control mice (Wilson et al. 2013). Previous studies with water deprivation for 3 days demonstrate increases of mRNA abundance of HSP‐70 (Cowley et al. 1995) and BGT1 (Moeckel et al. 1997) in the renal medulla. The difference could be due to the types of treatment (water deprivation vs water restriction), length of treatment (3 days vs. 28 h), or genetic alteration of non‐SCD mice (Paszty et al. 1997; Ryan et al. 1997). The absence of a significant effect of water restriction on NFAT5 in the non‐SCD inner medulla does not mean that NFAT5 is not important to concentration of urine under the “normal” condition. NFAT5 plays an indispensable role in protecting the renal medulla from hypertonic injury under this condition (Lopez‐Rodriguez et al. 2004).

### SCD mice could activate NFAT5 by inhibiting SHP‐1 in response to water restriction

NFAT5 is regulated by multiple mechanisms, including transactivation, DNA binding, nuclear localization and gene expression (Zhou 2016). More than a dozen of kinases have been demonstrated to contribute to tonicity‐dependent NFAT5 transactivation in cell culture (Zhou 2016). SHP‐1 is one of only a few signaling molecules that has an effect on NFAT5 nuclear localization in cultured cells (Zhou et al. 2010). SHP‐1‐S591, which is adjacent to the C‐terminal nuclear localization signal KRK, is pivotal in directing SHP‐1 cellular trafficking. It has been proposed that the negatively charged S591‐P interferes with the positively charged KRK function, resulting in reduced SHP‐1 nuclear localization (Tsui et al. 2006). We previously observed that in HEK293 cells high NaCl reduces SHP‐1 nuclear localization associated with increase of SHP‐1‐S591‐P, being consistent with the notion that SHP‐1‐S591‐P interferes with the KRK nuclear localization signal (Zhou et al. 2010). SHP‐1 reduces NFAT5 nuclear localization, and hypertonicity increases NFAT5 nuclear accumulation by expelling SHP‐1 from nuclei or restricting SHP‐1 from entering nuclei in HEK293 cells (Zhou et al. 2010). This mechanism was also observed in the SCD mice. Under the water replete condition, NFAT5 and SHP‐1 distributed between cytosol and nuclei. Water restriction increased NFAT5 nuclear accumulation and activity associated with increase of SHP‐1‐S591‐P and disappearance of SHP‐1 from nuclei to surrounding cytosol (Figs. 7 to 9). In addition to the increase in SHP‐1‐S591‐P, a reduction in water consumption also decreased SHP‐1 protein abundance in the SCD mouse inner medulla (Fig. 8C). This could be another mechanism for water restriction‐induced activation of NFAT5 in SCD.

In the non‐SCD mouse inner medulla, water restriction had no significant effect on NFAT5 nuclear localization and transcriptional activity (Fig. 7). Similarly, water restriction had no significant effects on SHP‐1‐S591‐P or SHP‐1 protein abundance (Fig. 8B), yet, it reduced SHP‐1 nuclear localization with increase of SHP‐1 presence in certain cytosolic region, which is different from the roughly even distribution of SHP‐1 in the cytosol in SCD mice. Whether this pattern is due to the lack of the effect of water restriction on SHP‐1‐S591‐P is unknown. Further, SCD itself had no significant effect on SHP‐1‐S591‐P or SHP‐1 protein abundance (Fig. 8A) despite that SCD reduced NFAT5 nuclear localization and transcriptional activity (Fig. 6), whereas knockout of ClC‐K1 channels reduced SHP‐1‐S591‐P and increased SHP‐1 protein abundance associated with inhibition of NFAT5 activity (Fig. 8D). SCD only reduced urinary osmolality by 17% under water repletion (Fig. 1A), whereas knockout of ClC‐K1 channels reduced urinary osmolality by 46% under a similar condition (Matsumura et al. 1999). It is possible that the urinary concentration defect induced by SCD is not severe enough to trigger the changes in SHP‐1‐S591‐P and SHP‐1 protein. It is also possible that SCD may already start to increase the SHP‐1 activity in the absence of showing alteration of SHP‐1‐S591‐P and SHP‐1 protein. Therefore, a definitive answer to whether SHP‐1 inhibits NFAT5 activity in the kidney inner medulla requires deletion of SHP‐1 from the region.

In summary, in contrast to the low levels of circulatory vasopressin and expression of AQP2 and NKCC2 in central diabetes insipidus and high levels of circulatory vasopressin and low levels of expression of AQP2, UTA1, and NKCC2 caused by nephrogenic diabetes insipidus, SCD‐induced urinary concentration defect is associated with elevated urinary vasopressin level and increased abundance of AQP2, UTA1, ENaC‐*β* subunit and possible NKCC2 protein and apical targeting of AQP2 in the mouse medulla. SCD mice strive to concentrate urine under the water replete condition in a vasopressin‐dependent manner. When water supply is reduced, the SCD mice activate NFAT5, but only modestly increase urinary osmolality. This struggle could be due to impaired countercurrent multiplication mechanism resulted from congestion of red blood cells in the vasa recta. SCD mice activate NFAT5 most likely by inhibiting SHP‐1 in response to water restriction.

## Disclaimer

The content and views expressed in this article are the sole responsibility of the authors and do not necessarily reflect the views or policies of the Department of Defense or US Government. Mention of trade names, commercial products, or organizations does not imply endorsement by the Department of Defense or U.S. Government.

## Conflict of Interest

None declared.
